# Effect of ascorbic acid and alpha-tocopherol supplementations on serum leptin, tumor necrosis factor alpha, and serum amyloid A levels in individuals with type 2 diabetes mellitus

**Published:** 2015

**Authors:** Mostafa Jamalan, Mahin Rezazadeh, Majid Zeinali, Mohammad Ali Ghaffari

**Affiliations:** 1 *Department of Biochemistry, School of Medicine, Ahvaz Jundishapur University of Medical Sciences, Ahvaz, Iran*; 2 *Biotechnology Research Center, Research Institute of Petroleum Industry (RIPI), Tehran, Iran*; 3 *Cellular and Molecular Research Center, Ahvaz Jundishapur University of Medical Sciences, Ahvaz, Iran*

**Keywords:** *Diabetes mellitus type 2 Ascorbic acid*, *Alpha-tocopherol*, *Leptin*, *Serum amyloid A*

## Abstract

**Objective:**

Diabetes mellitus Type 2 is one of the most widespread chronic metabolic diseases. In most cases, this type of diabetes is associated with alterations in levels of some inflammatory cytokines and hormones. Considering anti-inflammatory properties of plant extracts rich in ascorbic acid (vitamin C) and alpha-tocopherol (vitamin E), anti-diabetic properties of these two well-known antioxidant vitamins were investigated through measurement of serum levels of high-sensitivity C-reactive protein (hs-CRP), insulin, leptin, tumor necrosis factor alpha (TNF-α), and serum amyloid A (SAA) in patients with diabetes mellitus type 2.

**Materials and Methods:**

Male patients (n=80) were randomly divided into two groups each consisted of 40 subjects. Test groups were supplemented with ascorbic acid (1000 mg/day) or alpha-tocopherol (300 mg/day) orally during four weeks. Before and after treatment, serum biochemical factors of subjects were measured and compared.

**Results:**

Our results showed that both ascorbic acid and alpha-tocopherol could induce significant anti-inflammatory effects by decreasing the level of inflammatory factors such as TNF-α, SAA, and hs-CRP in diabetes mellitus type 2 patients. Effects of alpha-tocopherol and ascorbic acid in decreasing serum leptin level were similar. Ascorbic acid in contrast to alpha-tocopherol diminished fasting insulin and HOMA index but had no effect on LDL serum level.

**Conclusion:**

Concerning the obtained results, it is concluded that consumption of supplementary vitamins C and E could decrease induced inflammatory response in patients with diabetes mellitus type 2. It is also possible that vitamin C and vitamin E supplementation can attenuate incidence of some proposed pathological effects of diabetes mellitus**.**

## Introduction

Diabetes mellitus Type 2 is the most common form of diabetes and the eighth leading cause of death in the world. It is a chronic disease with a higher level of glucose in the blood of affected peoples (Duncan et al., 2003 ▶). More than 85% of people with type 2 diabetes are overweight. It is shown that visceral obesity could increase risk of metabolic diseases due to chronic inflammation. Dysregulated production of inflammatory cytokines (e.g., TNF-α and IL-6) by obese adipose tissues over the anti-inflammatory adipose tissue-derived humoral mediators (adipokines such as adiponectin) is known to induce a condition referred to as insulin resistance (Nishimura et al., 2009 ▶). Insulin resistance is a state in which a given concentration of insulin produces a less-than-expected biological effect. It is possible to control insulin resistance and diabetes by modulating inflammatory cytokines and adipokines using chemical drugs or supplementary micronutrients. Recently, it is suggested that deficiencies of some micronutrients are associated with obesity and related diseases (Garcia-Diaz et al., 2010 ▶). This relationship may be affected by leptin.

Leptin as an adipokine plays a key role in regulating energy intake and expenditure (Brennan and Mantzoros, 2006 ▶). Leptin is synthesized primarily in the adipocytes of the white adipose tissue and the level of circulating leptin is proportional to the total amount of fat in the body (Fischer et al., 2002 ▶). Although leptin mainly exert its effects through receptors in the hypothalamus (Williams et al., 2009 ▶), but it also has receptors on the pancreatic beta-cells for modulation of insulin expression in a negative feedback loop (Kieffer et al., 1996 ▶). Epidemiological studies have shown that the increased baseline leptin level in men is associated with increased risk of developing diabetes (McNeely et al., 1999 ▶; Tong et al., 2005 ▶). Vitamin C has been shown to inhibit leptin secretion and glucose uptake (Garcia-Diaz et al., 2010 ▶). Moreover, retinoic acid (Hollung et al., 2004 ▶; Felipe et al., 2005 ▶) and vitamin E (Zillikens et al., 2010) have been shown to decrease leptin expression and secretion.

There are evidences about involvement of inflammation in the pathophysiology of diabetes (Yudkin, 2003 ▶). TNF-α which is secreted by macrophages and a broad variety of cells including adipocytes (Gimeno and Klaman, 2005 ▶) is an adipocytokine involved in systemic inflammation (Moller, 2000 ▶). This cytokine inhibits insulin transduction and affects glucose metabolism (Zou and Shao, 2008). Association of TNF-α with insulin resistance in diabetes mellitus type 2 has been shown (Yudkin, 2003 ▶; Swaroop et al., 2012 ▶). Increased circulating concentration of TNF-α has been reported in patients with diabetes mellitus type 2 and impaired glucose tolerance (Pickup et al., 2000 ▶; Yudkin, 2003 ▶). 

Different isoforms of serum amyloid A (SAA) proteins are expressed in the liver in response to inflammatory stimuli. SAA proteins act as a cytokine, influencing cell adhesion, migration, and proliferation. Now, it is known that SAA may participate in the pathogenesis of chronic inflammatory diseases. It is shown that SAA proteins are increased in the plasma of obese and insulin resistant humans (Uhlar and Whitehead, 1999 ▶). Therefore, SAA is a potential target in the treatment of diseases associated with chronic inflammation including metabolic diseases. 

With regard to the above-mentioned facts, recognition of dietary supplements that are able to diminish serum levels of leptin and inflammatory factors such as TNF-α and SAA, could be important in the control of diabetic complications. Therefore, the objective of this study was to investigate the relationship between the serum levels of leptin, TNF-α, and SAA in diabetes mellitus type 2 with nutritional status of vitamins E and C. 

## Materials and Methods

**Materials**

Commercial glucose, triglyceride, cholesterol, HDL, LDL, and hs-CRP assay kits were obtained from Parsazmun Company (Tehran, Iran). Alpha-tocopherol, and ascorbic acid were purchased from Health (California, U.S.A). Commercial kits for measurements of insulin, leptin, TNF-α, and SAA protein were obtained from Diaplus Company (St. Louis, Mo, USA). All other reagents were purchased from Sigma (St. Louis, Mo, USA).

**Study subjects**

Iranian male subjects with type 2 diabetes (mean age: 52±8, mean BMI: 32±3, and mean duration of diabetes: 2±0.5 years) from Ahvaz Emam Hospital (Ahvaz, Iran) between March 2012 and April 2013, were selected for inclusion in our clinical study (IRCT201202208025N3). Subjects with kidney diseases, active infection, inflammatory diseases, hypo/hyperthyroidism, liver diseases, myocardial infarction and blood disorders, and those receiving medications containing diuretics or vitamins (C or E) were excluded from study. Diabetic subjects with hypertension (systolic blood pressure ≥ 140 mmHg) were also excluded from the study. The clinical trial protocol was reviewed and approved by the Institutional Ethics Committee of Ahvaz University of Medical Sciences, and the signed informed consent was obtained from all subjects. The selected subjects (n=80) were randomly divided into two groups each consisted of 40 subjects. The first group received ascorbic acid (1000 mg/day) and the other group, alpha-tocopherol (300 mg or 400 IU/day), orally during four weeks.

**Blood collection and biochemical analyses**

Before and after treatment of human subjects with ascorbic acid or alpha-tocopherol, blood samples were collected after an overnight fasting. Blood samples were centrifuged immediately at 4000 rpm for 5 minutes and then plasma fractions were separated and divided into aliquots. Serum levels of glucose, triglyceride, cholesterol, HDL, and LDL were immediately measured using a clinical chemistry analyzer (Olympus, AU400, Hamburg, Germany) and other samples were stored at -70 °C for further analysis of insulin, hsCRP, leptin, TNF-α, and SAA. Homeostasis assessment model was used to assess insulin resistance (HOMA.IR) using fasting insulin and glucose concentration by the following formula (Matthews et al., 1985 ▶).

HOMA-IR%=fasting serum glucose (mg/dL)×fasting insulin (μIU/mL)/405 

HOMA-IR<3 was considered as not-insulin resistance and HOMA-IR>5 was defined as insulin resistance (Miyazaki et al., 2003 ▶)

**Statistical analysis**

Data were expressed as mean ± SD. Significance of difference between means before and after treatments in each group was determined by paired student’s t-test. For comparison of two test groups with each other, independent (unpaired) student’s t-test was performed. P-values<0.05 were considered significant. 

## Results

**Effects of ascorbic acid and alpha-tocopherol supplementation on certain serum biochemical parameters of diabetic subjects**

In this study, diabetic patient subjects were randomly divided into two groups of 40 patients each and treated separately with ascorbic acid or alpha-tocopherol. Metabolic parameters of patients before and after administration of ascorbic acid or alpha-tocopherol are shown in Table 1. According to the obtained results, although treatment with ascorbic acid or alpha-tocopherol decreased fasting blood glucose (FBG), but this reduction was not statistically significant (Table 1). Neither ascorbic acid nor alpha-tocopherol supplementation could induce any significant effect on the levels of high-density lipoprotein (HDL) and triglyceride (TG) in the treated subjects. Oral administration of alpha-tocopherol decreased the levels of total cholesterol and low-density lipoprotein (LDL) in the treated group (Table 1). 

**Effects of ascorbic acid and alpha-tocopherol supplementation on the serum levels of hs-CRP and fasting insulin**

Our results demonstrated that oral administration of ascorbic acid or alpha-tocopherol in patients with type 2 diabetes could significantly diminish plasma level of hs-CRP (Table 1). 

Between various proposed approaches for quantitative assessment of insulin resistance and beta-cell function, HOMA-IR is the most suitable method for epidemiological studies. Our results showed that administration of alpha-tocopherol alone could not induce any detectable change on the fasting insulin or HOMA-IR% in type 2 diabetes patients. In contrast to alpha-tocopherol, our finding demonstrated the effectiveness of ascorbic acid in reducing fasting insulin and HOMA-IR% in type 2 diabetes patients (Table 1).

**Effects of alpha-tocopherol and ascorbic acid supplementation on leptin and TNF-α levels**

Oral administration of ascorbic acid in the diabetic patient subjects led to the decrease of fastening insulin and leptin while administration of alpha-tocopherol just diminished leptin concentration in patient subjects (Table 1). To our acknowledge, this is the first report about effects of ascorbic acid and alpha-tocopherol on leptin level in type 2 diabetes patients. 

Hyperglycemia due to type 2 diabetes induces higher levels of some inflammatory cytokines such as TNF-α. As seen in Table 1, both alpha-tocopherol and ascorbic acid could significantly diminish serum levels of TNF-α in patients with type 2 diabetes. 

**Table 1:** Metabolic parameters of subjects with type 2 diabetes before and after oral administration of ascorbic acid or alpha-tocopherol

		**Ascorbic acid group (n=40)**		** Alpha-tocopherol group (n=40) **	
		**Before treatment**	**After treatment**	**Before treatment**	**After treatment**
FBG (mg/dl)		181 ± 15	175 ± 12	198 ± 14	187 ± 16
Cholesterol (mg/dl)		185 ± 33	158 ± 29 *	223 ± 37	155 ± 29 *
LDL (mg/dl)		95 ± 27	92 ± 21	101 ± 23	81 ± 18 *
HDL (mg/dl)		56 ± 6	61 ± 11	54 ± 6	53 ± 5
TG (mg/dl)		157 ± 20	159 ± 38	153 ± 33	155 ± 35
hs-CRP (mg/dl)		4 ± 3	3 ± 2 *	3 ± 2	2 ± 1 *
HOMA-IR%		8 ± 3	3 ± 1 *	6 ± 2	5.5 ± 2
Fasting Insulin (µIU/ml)		17 ± 5	7 ± 3 *	13 ± 3	12 ± 3
Leptin (ng/ml)		31 ± 15	19 ± 12 *	57 ± 28	16 ± 8 *
TNF-alpha (ng/ml)	136 ± 20		82 ± 11 *	155 ± 25	41 ± 17 *
SAA (ng/ml)		169 ± 18	70 ± 16 *	115 ± 20	99 ± 15 *

Values are mean ± SD; n: number of subjects; FBG: fasting blood glucose; LDL: low density lipoprotein; HDL: high density lipoprotein; TG: triglyceride; hs-CRP: high-sensitivity C-reactive protein; HOMA-IR: homeostasis model of assessment-insulin resistance; TNF: tumor necrosis factor; SAA: serum amyloid A.

* : p< 0.05 was considered statistically significant.

**Effects of alpha-tocopherol and ascorbic acid supplementation on SAA level**

SAA protein as a well-known inflammatory marker and as an indicator of insulin resistance, decreased in diabetic subjects after treatment with ascorbic acid or alpha-tocopherol (Table 1).

## Discussion

Chronic inflammation is closely related to insulin resistance in type 2 diabetes (Bastard et al., 2006 ▶; Esser et al., 2014 ▶; Kaur, 2014 ▶). Therefore, with attention to extensive anti-inflammatory effects of alpha-tocopherol and ascorbic acid on downstream markers of inflammation, we used these vitamins for attenuation of inflammation in type 2 diabetes patients. 

There are some controversial reports about hypocholesterolemic effects of ascorbic acid (Myasnikov, 1958 ▶; Samuel and Shalchi, 1964 ▶). The works by Ginter et al. (Ginter et al., 1977 ▶) showed that the effect of vitamin C was dependent on the starting concentration of plasma cholesterol. A significant decrease in FBS, TG, LDL, HbA1C, and serum insulin was seen in diabetic patients supplemented daily with 1000 mg vitamin C for six weeks (Afkhami-Ardekani and Shojaoddiny-Ardekani, 2007 ▶). Meta-analysis of 13 randomized controlled trials by McRae (McRae, 2008 ▶) showed that supplementation with at least 500 mg vitamin C daily for a minimum of 4 weeks, can result in a significant decrease in serum LDL cholesterol and triglyceride concentrations but not a significant elevation in HDL cholesterol. Our results showed that ascorbic acid supplementation (1000 mg/day for four weeks) could not induce any significant effect on the levels of FBG, LDL, HDL, and also TG in the diabetic subjects. 

There are several reports about anti-peroxidative effects of vitamin E on LDL (Reaven et al., 1995 ▶; Fuller et al., 2000 ▶) but its effects on the serum levels of FBG, LDL, HDL, or TG especially in diabetic patients is less studied. We showed that in contrast to vitamin C, alpha-tocopherol could significantly lower total serum cholesterol and LDL in the treated subjects. 

Increasing level of hs-CRP as an inflammatory marker is associated with higher risk of cardio-vascular diseases (CVD). Furthermore, elevated level of hs-CRP is associated with ischemic stroke and death from severe kinds of cancers and lung diseases (Kaptoge et al., 2010 ▶). Moreover, hs-CRP is recommended as a predictive laboratory marker for CVD risk in patients with diabetes mellitus (Haffner, 2006 ▶). With regards to the higher risk of CVD in persons with diabetes mellitus (nearly two folds in comparison to healthy ones), control of inflammatory factors such as hs-CRP is critical (Sarwar et al., 2010 ▶). It has been shown that ascorbic acid, as a major antioxidant, could be effective in the reduction of hs-CRP level and consequent suppression of inflammation in patients undergoing hemodialysis (Zhang et al., 2011; Biniaz et al., 2014 ▶). Moreover, alpha-tocopherol exerts anti-inflammatory effects through a number of different mechanisms, for example, by decreasing levels of CRP and pro-inflammatory cytokines as well as by inhibiting the activity of protein kinase C and other enzymes, such as cyclooxygenase-2 (Singh et al., 2005 ▶; Calder et al., 2009 ▶). Our results (Table 1), in confirmation with previous studies by Devaraj and Jialal (Devaraj and Jialal, 2000 ▶) and in contradiction with the work (Wu et al., 2007 ▶), demonstrated that alpha-tocopherol could reduce plasma level of hs-CRP in diabetic patients. In a similar manner, ascorbic acid was also able to decrease hs-CRP level significantly.

 Between various proposed approaches for quantitative assessment of insulin resistance and beta-cell function, HOMA-IR is the most suitable method for epidemiological studies (Wallace and Matthews, 2002 ▶). Positive effect of antioxidant supplementation on HOMA-IR index has been shown in healthy individuals (Vincent et al., 2009 ▶). In addition, Lai (Lai, 2008 ▶) has shown that co-administration of alpha-tocopherol or ascorbic acid with chromium could decrease HOMA index and improve glucose metabolism in type 2 diabetic patients. On the other hand, it has been shown that administration of alpha-tocopherol alone could not induce any detectable change on HOMA-IR% and fasting insulin in type 2 diabetic patients (Shadman et al., 2013 ▶), a conclusion which is confirmed with our results in the current study (Table 1). In contrast with previous work by Lai (2008), ▶ our finding demonstrated the effectiveness of ascorbic acid alone in reducing fasting insulin and HOMA-IR% in type 2 diabetes patients. 

Leptin is a peptide hormone which is released by adipocytes and could inhibit obesity by stimulating satiety centers in brain (DePaoli, 2014 ▶). Most of obese peoples exhibit leptin receptor deficiency, which consequently lead to leptin resistance condition (Tartaglia et al., 1995 ▶). The works by Fischer et al. (Fischer et al., 2002 ▶) showed that leptin level in patients with type 2 diabetes is higher than normal. They confirmed a positive correlation between fasting leptin level and insulin resistance independent of body fat mass. We showed for the first time that oral administration of alpha-tocopherol or ascorbic acid could decrease serum leptin level in diabetic subjects.

Elevation of TNF-α concentration in patients with diabetes mellitus type 2 and impaired glucose tolerance has been reported in different investigations (Pickup et al., 2000 ▶; Yudkin, 2003 ▶). Hyperglycemia due to type 2 diabetes induces higher levels of some inflammatory cytokines such as TNF-α through down-regulation of CD33 in primary human monocytes (Gonzalez et al., 2012 ▶). In an in vitro study, it was shown by Gonzalez et al. (Gonzalez et al., 2012 ▶) that alpha-tocopherol inhibits TNF-α production by monocytes at high-glucose concentrations. In another work by Chen et al. (Chen et al., 2014 ▶), it was shown that ascorbic acid could inhibit LPS-induced TNF-α in vitro. Our results in accordance with in vitro studies showed that both ascorbic acid and alpha-tocopherol could decrease serum level of TNF-α in patients with diabetes mellitus type 2.

SAA protein as a well-known inflammatory marker and as an indicator of insulin resistance (Rho et al., 2009 ▶; Gonzalez et al., 2012 ▶) has positive correlation with obesity (Scheja et al., 2008). As stated earlier in the introduction section, it is shown that the level of SAA proteins in the plasma of obese and insulin resistant humans is higher compared with healthy ones (Uhlar and Whitehead, 1999 ▶). Our finding showed that consumption of ascorbic acid or alpha-tocopherol could diminish level of SAA proteins in diabetic subjects. 

In conclusion, according to the obtained results, it seems that ascorbic acid and alpha-tocopherol could induce inhibitory effects on inflammatory markers such as SAA, TNF-α, and leptin. Therefore, oral consumption of ascorbic acid and alpha-tocopherol as anti-inflammatory agents could be beneficial for decreasing inflammation in type 2 diabetes patients. 
